# Specificity and Durability of Changes in Auditory Processing Efficiency After Targeted Cognitive Training in Individuals With Recent-Onset Psychosis

**DOI:** 10.3389/fpsyt.2020.00857

**Published:** 2020-08-28

**Authors:** Bruno Biagianti, Melissa Fisher, Rachel Loewy, Benjamin Brandrett, Catalina Ordorica, Kristin LaCross, Brandon Schermitzler, Michelle McDonald, Ian Ramsay, Sophia Vinogradov

**Affiliations:** ^1^ Department of R&D, Posit Science Corporation, San Francisco, CA, United States; ^2^ Department of Pathophysiology and Transplantation, University of Milan, Milan, Italy; ^3^ Department of Psychiatry and Behavioral Sciences, University of Minnesota, Minneapolis, MN, United States; ^4^ Department of Psychiatry, University of California, San Francisco, San Francisco, CA, United States; ^5^ Division of Psychology & Language Sciences, University College London

**Keywords:** cognitive training, neuroplasticity, target engagement, early psychosis, personalized medicine

## Abstract

**Background:**

We previously demonstrated that the high heterogeneity of response to computerized Auditory Training (AT) in psychosis can be ascribed to individual differences in sensory processing efficiency and neural plasticity. In particular, we showed that Auditory Processing Speed (APS) serves as a behavioral measure of target engagement, with faster speed predicting greater transfer effects to untrained cognitive domains. Here, we investigate whether the ability of APS to function as a proxy for target engagement is unique to AT, or if it applies to other training interventions, such as Executive Functioning Training (EFT). Additionally, we examine whether changes in APS are durable after these two forms of training.

**Methods:**

One hundred and twenty-five participants with Recent Onset Psychosis (ROP) were randomized to AT (n = 66) and EFT (n = 59), respectively. APS was captured at baseline, after treatment, and at 6-month follow-up. Mixed models repeated measures analysis with restricted maximum likelihood was used to examine whether training condition differentiated APS trajectories. Within-group correlational analyses were used to study the relationship between APS and performance improvements in each of the training exercises.

**Results:**

The two groups were matched for age, gender, education, and baseline APS. Participants showed high inter-individual variability in APS at each time point. The mixed model showed a significant effect of time (F = 5.99, *p* = .003) but not a significant *group-by-time* effect (F = .73, *p* = .48). This was driven by significant APS improvements AT patients after treatment (d = .75) that were maintained after 6 months (d = .63). Conversely, in EFT patients, APS improvements did not reach statistical significance after treatment (p = .33) or after 6 months (p = .24). In AT patients, baseline APS (but not APS change) highly predicted peak performance for each training exercise (all *r’*s >.42).

**Conclusions:**

Participant-specific speed in processing basic auditory stimuli greatly varies in ROP, and strongly influences the magnitude of response to auditory but not executive functioning training. Importantly, enhanced auditory processing efficiency persists 6 months after AT, suggesting the durability of neuroplasticity processes induced by this form of training. Future studies should aim to identify markers of target engagement and durability for cognitive training interventions that target sensory modalities beyond the auditory domain.

## Introduction

Uncoordinated neural activity during early sensory processing is well-documented in individuals with schizophrenia early in the course of illness (1–3), and among individuals at risk for developing psychosis (4–6). Impaired early processing operations are known to contribute to widespread neurocognitive-perceptual impairments, including deficits in attention, speed of processing, learning and memory, problem solving, and executive functioning (7). Albeit heterogeneous, these impairments are observed across the illness course of schizophrenia (8), are present in antipsychotic-naïve first-episode individuals and in individuals at risk for psychosis (9, 10), and predict the transition from prodromal to first-episode psychosis (11). Therefore, sensory processing has evolved as a target for experimental interventions aiming to remediate schizophrenia-related cognitive impairments, including computerized cognitive training (12).

The computerized training program that has been most consistently studied to date to target sensory processing in the auditory system was developed by Posit Science, Inc. (13). Via exercises that place implicit, increasing demands on discrimination of basic auditory and verbal stimuli, Auditory Training (AT) is designed to “re-tune” the operations between temporally detailed resolution of auditory inputs in auditory cortex, prefrontally-mediated attention, and auditory/verbal memory functions (14). Indeed, electro- and magneto-encephalographic data suggest that AT enhances both early representations in primary auditory cortex and auditory sensory gating, as well as both early and later task-related activity in prefrontal areas (3, 15–18).

According to its hypothesized mechanism of action, as well as neurophysiological findings from studies investigating its neural underpinnings (17, 19), AT drives gains in higher-order cognitive operations by improving efficiency in distributed prefrontal-temporal auditory systems (20, 21). Because several lines of evidence suggest that the degree of engagement of targeted neural systems can be indexed by behavioral changes on a neurophysiological task specifically designed to probe such systems (13, 18, 22–25), we sought to identify and characterize a behavioral measure of sensory processing efficiency, indicative of both auditory perceptual and attentional operations.

In our previous work, we showed that Auditory Processing Speed (APS): (i) can be reliably measured in patients with schizophrenia undergoing AT *via* a time-order judgment task using frequency-modulated sound stimuli (21, 26, 27); (ii) significantly improves after 20 h of AT (21, 28, 29), but does not show additional significant changes at 30 or 40 h of training (21, 30); and (iii) shows a high degree of inter-individual variability at baseline, in the steepness of the initial change, and in the efficiency threshold reached after 20 h—indicating heterogeneity both in terms of baseline psychophysical efficiency and in terms of training-induced engagement of prefrontal-temporal neural systems (21). Most importantly, we found that the faster the APS that is reached after 20 h of training (and not the overall magnitude of APS improvements), the greater the transfer effects to untrained cognitive domains—suggesting a significant association between a patient’s ability to reach a threshold of sensory processing efficiency and their degree of cognitive improvement after AT (21).

In light of such findings, three questions emerge as the logical next steps of investigation. First, because the behavioral assessment of sensory processing to date has only been applied to the study of AT, it remains unclear whether APS can capture the enhancement of processing efficiency induced by training programs targeting sensory modalities other than the auditory domain. As novel computerized training interventions become increasingly available, including Social cognitive Training (31–34), and Executive Functioning Training (EFT; 31, 34, 35), it is critical to evaluate whether APS can still serve as a proxy of neural target engagement, or if new behavioral measures need to be developed and validated.

Second, the idea that improved auditory system processing translates to enhanced cognitive performance needs to be further verified, as results on the relationship between improvements on the AT exercises, APS, and gains in cognitive outcomes have been somewhat inconsistent. In fact, three studies reported significant associations between APS and cognitive outcomes (21, 26, 27), whereas two found substantial gains in APS, but no transfer of gains to other cognitive outcome measures (28, 29). A significant relationship between APS and improvements on the AT exercises would corroborate its ability to serve as a measure of target engagement.

Third, while the efficacy of AT for improving cognition and neural activation patterns in schizophrenia in the short term is well supported (16, 26, 27, 36), as well as its capacity to induce durable improvements in neuropsychological and functional outcome measures (18, 31, 37), no studies to date have investigated the durability of improvements in processing efficiency following AT or other forms of computerized training.

To address these questions, we collected data from patients with Recent Onset Psychosis (ROP) who were randomized to AT and EFT, and measured APS at baseline, immediately after treatment, and at 6-month follow-up.

## Methods

### Participants

Study participants consisted of 125 adolescents and young adults recruited in the context of an ongoing, double-blind, multi-site randomized controlled trial (ClinicalTrials.gov Identifier: NCT01973270) that was performed across four community mental health centers in California (Prevention and Recovery in Early Psychosis, PREP) and from the University of Minnesota Physicians Psychiatry (UMP) Outpatient Clinics, each of which specialize in early intervention services following the NAVIGATE model (38). Participants receiving treatment services were referred by their clinicians or were recruited by the study team.

They met criteria for a DSM-V Psychotic Disorder, with onset of first psychotic episode within the last 2 years. All participants had achieved outpatient status for at least 1 month before study entry and participants taking psychiatric medications were on a stable dose for at least 1 month prior to participation. All participants met the following additional inclusion/exclusion criteria: (1) Good general physical health; (2) Age 16–35 years; (3) Fluent and proficient in English; (4) IQ ≥70; (5) No neurological disorder; (6) No clinically significant substance abuse that would interfere with the ability to participate fully during recruitment, assessment, or training; (7) Not being currently treated with benztropine, diphenhydramine, or high doses of clozapine (>500 mg by mouth four times a day) or olanzapine; (8) No prior cognitive training within the past 3 years.

### Procedures

Participants age 18 and older gave written informed consent, while those younger than age 18 provided assent, with written parental/legal guardian consent. Baseline assessments were conducted prior to randomization. Participants were randomly assigned to two types of cognitive training: AT and Social Cognition Training (SCT), or EFT. Participants were loaned tablets and participated in the intervention at home or in the clinic. Participants were contacted 1–2 times per week by telephone, email, text, or during brief in-person meetings to discuss progress. Coaching was provided if a participant indicated difficulty in completing the recommended number of hours of training per week (e.g., goal-setting; discussion of scheduling; setting an alarm and using reminders). Participants were asked to complete up to 30 h of training (1 h/day, 5 days/week, for 6 weeks), and had up to 12 weeks to complete training prior to post-training assessments. Six months after training, participants completed the follow-up assessment.

While in the trial, participants received early intervention services by providers or clinic personnel not involved in the study (e.g., individual, group, and family therapy, case management, psychosocial rehabilitation, psychosocial education, psychiatric services, peer support services, and supportive employment and education services). All participants received $25 for every 5 h of training completed, as well as compensation for completing the baseline, post-training, and 6-month follow-up assessments.

Demographic characteristics are presented in **Table 1**.

**Table 1:** Demographics of participants randomized to Auditory Training (AT) and Executive Functioning Training (EFT).

	**AT (N = 66)Mean (SD)**	**EFT (N = 59)Mean (SD)**	**T-test (p-value)**
Female(N)/Male(N)/Other(N)^a^	14/46/6	15/44/0	.07 (.79)
Age (range 16–35)	21.5 (3.6)	21.3 (3.2)	.23 (.82)
Years of Education	13.0 (1.9)	12.8 (1.5)	.54 (.59)
Diagnosis Schizophrenia(N) Schizoaffective Disorder(N) Schizophreniform(N) Other Psychotic Disorders(N) Unspecified Schizophrenia Spectrum Disorder(N)	32 12 6 1 15	24 7 4 7 17	7.11 (.13)
Baseline Positive and Negative Syndrome Scale Total	61.80 (17.44)	62.67 (16.70)	.28 (.78)
Baseline APS (ms)	166 (309)	151 (302)	.35 (.73)
Post-Intervention APS (ms)	75 (269)	114 (269)	-1.52 (.13)
6-month APS (ms)	87 (316)	107 (251)	-.76 (.45)
Blocks of Computerized Training	338.4 (268.1)	327.1 (275.4)	.23 (.82)

a Chi-Square Test Results.

#### Cognitive Training Interventions

Participants in the targeted AT program completed auditory training and social cognition training exercises. AT has been described in detail previously (21). It consists of four computerized exercises (Sound Sweeps, Fine Tuning, Syllable Stacks, Fine Tuning) designed to improve speed and accuracy of auditory information processing while engaging auditory and verbal working memory. More information about the exercises can be found at https://www.brainhq.com/why-brainhq/about-the-brainhq-exercises. This training approach is based on evidence that schizophrenia is characterized by widespread disturbances in fronto-temporal neural systems subserving auditory processing and verbal memory. The targeted SCT program has been recently studied as a supplement to AT (31, 34). It consists of seven computerized exercises which collectively target perception, attention and memory in the social cognitive domains of vocal and visual affect perception and social cue perception (gazes and faces).

The targeted EFT program consists of eleven computerized exercises that engage visual attention and working memory processes to improve cognitive control. All exercises were provided by Posit Science Corporation.

All three training programs are structured in blocks. Each block consists of 20–50 adaptive trials. The difficulty level of each trial depends on performance on previous trials. Block completion is based on user performance: once exercise-specific algorithms detect lack of additional improvements, the block terminates, and users are presented with a new block from the same exercise. Therefore, even if the length of a training session is fixed (60 minutes) and the number of exercises within a session is fixed (4 exercises), different individuals complete different amounts of blocks per exercise within a session. Correct trials are rewarded with points and animations. Compliance is monitored by electronic data upload.

Four metrics are available for each exercise: (1) baseline performance – this is the score reached the first time a participant played with any given exercise; (2) number of blocks – this is a direct measure of exposure to a training exercise; (3) peak performance – this is the best score reached in a training exercise at any point throughout the intervention; 4) weighted peak performance – this is a weighted average of peak performance that takes into account the number of blocks completed for that specific exercise, such that the more blocks a participant has completed for a specific exercise, the more his peak performance weighs.

#### Assessment of Auditory Processing Speed (APS)

APS was determined using a method based on the Zippy Estimation by Sequential Testing (ZEST) algorithm, an adaptive Bayesian procedure for determining sensitivity measures (i.e. estimating threshold). In the assessment of APS, participants perform a time-order judgment task where they are asked to identify the direction of tonal change in a sequence of two successive frequency-modulated (FM) sound sweeps, as either “up” (from a lower to a higher pitch) or “down” (from a higher to a lower pitch). The ZEST algorithm adaptively modifies the interstimulus interval (ISI) between the two sound sweeps and the sweep duration, which is held equal to the ISI, as the performance changes trial by trial. The Bayesian procedure terminates after 100 trials.

Lower APS indicates faster (more efficient) auditory processing speed at shorter sweep durations. The task can be accessed *via* the url: https://v4.brainhq.com/?v4=true&fr=y#assessment/0N. APS was measured at baseline, immediately after training, and at 6-month follow-up.

#### Planned Analyses

We performed an intent-to-treat analysis on all randomized participants (*N* = 125), regardless of hours of intervention. Independent samples T-tests were used to test for group differences in age, participant education, and baseline performance in the APS task. Pearson’s chi-square was used to test for group differences in gender. Fisher’s Exact Test was used to test for group differences in medication regimens. In order to examine whether changes in APS are durable after training and whether training condition differentiates APS trajectories over time, we used a linear mixed-effects model with group and time as fixed factors. Model parameters were estimated using restricted maximum likelihood and AR (1) correlation structure. This allowed us to retain all available data for analysis. Effect sizes (Cohen’s *d*) were computed using the mean change scores (post-training minus baseline) and the baseline pooled standard deviations. To investigate whether metrics from the AT exercises were associated with APS, we used Pearson’s correlations uncorrected for multiple comparisons.

## Results

All variables were normally distributed. There were no significant differences between groups in baseline demographic characteristics, medication regimens, symptom severity, baseline APS, or total amount of training.

After randomization, 17 out of 66 (26%) AT participants withdrew from the study compared to 14 of 59 (24%) EFT participants, a nonsignificant difference (*χ* ^2^ = 0.08, *p* = 0.80). There were no significant differences in demographic variables and symptom severity between those who completed the training and those who dropped out (all *p* values >.05). At the 6-month follow-up, 34 AT participants and 21 EFT participants completed the APS assessment, respectively. There were no baseline differences in demographic variables, baseline APS, and symptom severity between participants who dropped out at the post-training time point versus six-month follow up completers (all *p* values >.05).

At each time point (baseline, post intervention, 6 months), there was a high degree of interindividual variability for APS (see SDs in **Table 1**). There were significant main effects of time (F = 5.99, *p* = .003), but not significant *group-by-time* effects (F = .73, *p* = .48). AT patients showed significant APS improvements after treatment (*d* = .75, *p* < 0.05) that were significantly sustained after 6 months (baseline to six-month *d* = .63, *p* < 0.05). Although EFT showed APS improvements, such improvements did not reach statistical significance after treatment (*d* = .13, *p* = .33) or after 6 months (*d* = .16, *p* = .24).

In AT patients, baseline APS (but not APS change) highly predicted weighted peak performance for each training exercise, in that participants with better processing speed at baseline reached better peak performance during training (see **Table 2**).

**Table 2:** Pearson’s Correlations between APS and performance metrics on the AT exercises.

	**Weighted peak performance**	**Fine Tuning**	**Memory Grid**	**Sound Sweeps**	**Syllable Stacks**
**Baseline APS**	Pearson Correlation	.647	.716	.490	.422
	Sig. (2-tailed)	.003	.001	.033	.016
**Post-Intervention APS**	Pearson Correlation	.678	.350	.525	.254
	Sig. (2-tailed)	.015	.265	.080	.325
**6-month APS**	Pearson Correlation	.867	.441	.659	.193
	Sig. (2-tailed)	.000	.151	.020	.355
**APS change (Post-Intervention - Baseline)**	Pearson Correlation	.087	-.713	.176	-.461
	Sig. (2-tailed)	.853	.072	.705	.153

## Discussion

To our knowledge, this is the first study to generate valuable data on the relative effects on processing efficiency of two distinct cognitive training approaches for ROP—a targeted “distributed neural system” training model derived from systems neuroscience vs. a “general cognitive stimulation” training model derived from neuropsychological rehabilitation approaches.

We compared gains in APS between two different forms of cognitive training from baseline, to post-training, to 6 months after training. Our results showed a significant main effect of time driven by improvements in APS in the AT group in the medium to large range, and non-significant gains in the EFT group. These results suggest the specificity of APS as a proxy measure of target engagement of auditory processing since only the AT group showed significant improvements post-training, and durability of these improvements 6 months after training.

Nonetheless, APS seems to capture an incremental improvement, albeit statistically non-significant, of processing efficiency (151 ms at baseline, 114 ms at post-intervention, 107 ms at 6-month) that is induced in participants undergoing EFT. While processing speed and executive functioning have been historically defined as two distinct neuropsychological constructs, so that AT and EFT are two cognitive training approaches targeting different neural systems and sensory modalities, findings of similar or almost identical networks underlying processing speed and executive functioning suggest that there could indeed be shared variance between these two constructs (39). Our hypothesis—in accordance with the hierarchical model for which impairments in executive functioning in ROP are largely influenced by processing speed impairments (40, 41)—is that EFT indirectly requires and targets processing speed functions that are systematically engaged when encoding stimuli during the training exercises.

In this context, and in line with recent evidence of significant relationships between auditory and visual processing speeds (42, Ramsay et al., *in this issue*), APS could be capturing only the fraction of variance in processing efficiency that is independent of sensory modality. Therefore, developing behavioral measures that are sensitive to the neural changes induced by EFT could be useful in consolidating its mechanism of action and efficacy. Ultimately, we imagine that a data-driven personalized combination of these two training approaches is likely to induce the greatest cognitive gains among the largest number of individuals (43).

In the AT group, baseline APS performance was strongly associated with weighted peak performance on all 4 of the AT exercises, indicating that baseline processing speed might be a useful indicator of the magnitude of learning. This is consistent with findings from Perez and colleagues, who showed in a sample of individuals with schizophrenia that better mismatch negativity—an event-related potential indexing pre-attentional auditory sensory discrimination—at baseline predicted greater performance after one hour of exposure to Sound Sweeps, one of the AT exercises (44). This suggests that it is possible to ascribe the high heterogeneity of response seen for AT to individual differences in auditory processing efficiency and sensory system “learning potential” that characterize schizophrenia.

While our results are promising in showing the specificity and durability of APS improvements after AT, and the relationship of this proxy measure of target engagement to performance on AT exercises, there are several limitations to this study. First, while significant gains in APS were seen only in the AT group, the omnibus group-by-time interaction did not reach statistical significance. These results should be interpreted with caution and require replication. Second, we did not examine the relationship of APS to changes in cognitive, symptom, or functional outcomes as this is an ongoing randomized controlled trial. These outcomes will be analyzed at the completion of the study.

Although our findings confirm the ability of APS to serve as a predictor of learning during the exercises (21, 27), ultimately indicating which individuals are likely to benefit from AT, the high degree of variability shown in APS calls for an individualized rather than a “one-size-fits-all” approach to cognitive training. Implementing AT without knowledge about individual variation in domains of brain function that influence therapeutic response continues to be problematic, with high rates of treatment non-responders (21, 34).

As a field, we need to continue to: (i) identify more baseline predictors of response to AT, in order to select patients that will be sensitive to this intervention; (ii) identify more markers of early target engagement, i.e. indices that will help us determine which neural and cognitive systems need to be critically engaged in order to induce cognitive gains following longer therapeutic protocols; (iii) identify more mediators of treatment response to AT that could be used in future fast-fail approaches to quickly determine treatment uptake for a given individual; (iv) identify more biomarkers of change, i.e. indices whose changes are significantly associated with psychophysical “learning” during AT as well as with improvements real-world outcomes after training. These avenues of investigation will promote an in-depth characterization of the mechanisms of action of cognitive training, allowing for a data-driven optimization and refinement of this promising treatment.

## Data Availability Statement

The datasets generated for this study are available on request to the corresponding author.

## Ethics Statement

The studies involving human participants were reviewed and approved by the Institutional Review Board (IRB) of the University of California San Francisco and the IRB of the University of Minnesota Twin Cities. Participants age 18 and older gave written informed consent, while those younger than age 18 provided assent, with written parental/legal guardian consent.

## Author Contributions

BB was responsible for statistical analysis, data interpretation, drafted the manuscript, and oversaw data collection. MF, RL, and IR assisted in statistical analysis and data interpretation. BBr, CO, KL, MM, and BS assisted in data collection and data entry. MF, SV, BB, and RL conceptualized and oversaw data collection. All authors contributed to the article and approved the submitted version.

## Funding

This study was supported by the National Institute of Mental Health under Award Numbers R43 MH121209-0 (PI: BB), U01MH108150 (PI: SS), R01MH102063-01 (PI: SS, RL), The Wells Family Foundation (PI: SV). The content is solely the responsibility of the authors and does not necessarily represent the official views of the funding agencies.

## Conflict of Interest

BB is Senior Scientist at Posit Science, a company that produces cognitive training and assessment software. The training programs described in this study were provided for research purposes free of charge by Posit Science.

The remaining authors declare that the research was conducted in the absence of any commercial or financial relationships that could be construed as a potential conflict of interest.

## Supplementary Material

Supplementary Table 1 Medication Regimens of Study Participants.
