# Supplementary material for: Specificity and Durability of Changes in Auditory Processing Efficiency After Targeted Cognitive Training in Individuals With Recent-Onset Psychosis
Source: Front Psychiatry. 2020 Aug 28;11:857. doi: 10.3389/fpsyt.2020.00857 (PMC7484996; doi:10.3389/fpsyt.2020.00857)
Supplement: Supplementary Table 1 — Medication Regimens of Study Participants. [file Table_1.docx]

**Supplementary Table 1.** Medication Regimens of Study Participants.

|  | TCT  Yes / No | GCE  Yes / No | Total^1^ | Fisher’s Exact Test p Value |
| --- | --- | --- | --- | --- |
| **Psychiatric Medication (N)** | 53/8 | 48/5 | 114 | 0.57 |
| **Antipsychotic Medication (N)** | 49/12 | 45/8 | 114 | 0.62 |
| 1^st^ Generation (N) | 0/61 | 2/51 | 114 | 0.21 |
| 2^nd^ Generation (N) | 49/12 | 44/9 | 114 | 0.81 |
| Multiple Antipsychotics (N) | 9/52 | 8/45 | 114 | 1.00 |
| Other Psychiatric Medication | | | | |
| Antidepressants | 24/37 | 18/35 | 114 | 0.57 |
| Mood Stabilizers | 7/54 | 9/44 | 114 | 0.43 |
| Anti-Anxiety  (non-Benzodiazepine) | 5/56 | 5/48 | 114 | 1.00 |
| Benzodiazepine | 10/51 | 5/48 | 114 | 0.41 |

^1^Medication information was unavailable for 11 of the 125 participants.
